# Targeting MUC1-C suppresses BCL2A1 in triple-negative breast cancer

**DOI:** 10.1038/s41392-018-0013-x

**Published:** 2018-05-12

**Authors:** Masayuki Hiraki, Takahiro Maeda, Neha Mehrotra, Caining Jin, Maroof Alam, Audrey Bouillez, Tsuyoshi Hata, Ashujit Tagde, Amy Keating, Surender Kharbanda, Harpal Singh, Donald Kufe

**Affiliations:** 1 Dana-Farber Cancer Institute, Harvard Medical School, Boston, MA USA; 2 Center for Biomedical, Indian Institute of Technology, Delhi, India; 3 Departments of Biology and Biological Engineering, Massachusetts Institute of Technology, Cambridge, MA USA; 4 Present Address: Department of Gastrointestinal Surgery, Graduate School of Medicine, Osaka University, Suita, Osaka, 565-0871 Japan

## Abstract

B-cell lymphoma 2-related protein A1 (BCL2A1) is a member of the BCL-2 family of anti-apoptotic proteins that confers resistance to treatment with anti-cancer drugs; however, there are presently no agents that target BCL2A1. The MUC1-C oncoprotein is aberrantly expressed in triple-negative breast cancer (TNBC) cells, induces the epithelial–mesenchymal transition (EMT) and promotes anti-cancer drug resistance. The present study demonstrates that targeting MUC1-C genetically and pharmacologically in TNBC cells results in the downregulation of BCL2A1 expression. The results show that MUC1-C activates the *BCL2A1* gene by an NF-κB p65-mediated mechanism, linking this pathway with the induction of EMT. The MCL-1 anti-apoptotic protein is also of importance for the survival of TNBC cells and is an attractive target for drug development. We found that inhibiting MCL-1 with the highly specific MS1 peptide results in the activation of the MUC1-C→NF-κB→BCL2A1 pathway. In addition, selection of TNBC cells for resistance to ABT-737, which inhibits BCL-2, BCL-xL and BCL-W but not MCL-1 or BCL2A1, is associated with the upregulation of MUC1-C and BCL2A1 expression. Targeting MUC1-C in ABT-737-resistant TNBC cells suppresses BCL2A1 and induces death, which is of potential therapeutic importance. These findings indicate that MUC1-C is a target for the treatment of TNBCs unresponsive to agents that inhibit anti-apoptotic members of the BCL-2 family.

## Treatment opportunities for tricky tumors

New insights into gene regulation reveal vulnerabilities in a particularly hard-to-treat form of breast cancer. Patients with the ‘triple-negative’ form of this disease have a particularly poor prognosis, and researchers led by Donald Kufe at the Dana-Farber Cancer Institute in Boston, USA recently investigated the role of the mucin 1 protein in such malignancies. Mucin 1 promotes the survival and aggressive growth of tumor cells, and the researchers identified multiple downstream genes that mediate these effects. Through these pathways, mucin 1 helps establish resistance to multiple different therapeutic agents. However, Kufe’s team determined that GO-203, a drug currently in early clinical development, can effectively shut down these mucin 1-activated signaling pathways in cultured breast cancer cells. The researchers are now moving this agent into clinical trials to see whether triple-negative patients might benefit.

## Introduction

Mucin 1 (MUC1) is a heterodimeric protein that is overexpressed in ~90% of triple-negative breast cancers (TNBCs).^1,2^ The MUC1 transmembrane C-terminal (MUC1-C) subunit functions as an oncoprotein by interacting with diverse kinases and effectors that have been linked to transformation.^1,3^ Along these lines, MUC1-C activates the inflammatory TAK1, TGF-β-activated kinase 1 (TAK1)→IKK→NF-κB p65 pathway.^4–6^ The MUC1-C cytoplasmic domain interacts directly with NF-κB p65 and promotes the activation of NF-κB p65 target genes, including *MUC1*, in an autoinductive loop that increases MUC1-C expression.^6^ Activation of MUC1-C→NF-κB p65 signaling is, in turn, associated with induction of the *ZEB1* gene, which encodes a transcriptional repressor that suppresses *miR-200c* and drives the epithelial–mesenchymal transition (EMT).^7^ MUC1-C also contributes to the loss of epithelial cell polarity by (i) ZEB1-mediated downregulation of the CRB3, HUGL2 and PATJ polarity factors and (ii) repression of the *CDH1* gene and thereby the expression of E-cadherin, a key protein for the formation of adherens junctions.^7–9^ EMT is a complex process that involves the recruitment of EMT-inducing factors, such as ZEB1, to key target genes in association with changes in their epigenetic regulation.^10^ In this context, MUC1-C→NF-κB p65 signaling activates transcription of the *DNA methyltransferase 1* (*DNMT1*) and *3b* (*DNMT3b*) genes and induces DNA methylation of the *CDH1* promoter, which suppresses E-cadherin expression.^11^ Emerging evidence supports a role for the MUC1-C→NF-κB p65 pathway in integrating EMT, epigenetic programming and a gene signature of immune evasion.^12,13^

The EMT program includes the acquisition of mesenchymal and stem cell traits with an increased capacity for survival.^10,14^ In this regard, MUC1-C has been linked to signals that attenuate stress-induced cell death.^1,3^ MUC1-C is transported to the mitochondrial outer membrane, where it blocks the apoptotic response to oxidative stress and DNA damage-induced stress.^15–17^ Mechanistically, MUC1-C binds to the pro-apoptotic BAX protein at the critical BH3 domain, thereby blocking BAX dimerization and the BAX-mediated release of mitochondrial cytochrome c.^18^ MUC1-C also increases the expression of the anti-apoptotic BCL-xL protein by an NF-κB p65-dependent mechanism^6^ and myeloid cell leukemia-1 (MCL-1) protein, which is a major cause of drug resistance in TNBC cells.^19^ B-cell lymphoma 2-related protein A1 (BCL2A1) is another member of the BCL-2 family of anti-apoptotic proteins that is associated with resistance to chemotherapeutics and targeted agents.^20^ *BCL2A1* functions as a lineage-specific oncogene by blocking cell death.^21,22^ However, there are presently no effective agents for the treatment of cancers that overexpress BCL2A1.

There is no known relationship between MUC1-C signaling and BCL2A1. The present study demonstrates that MUC1-C induces BCL2A1 expression in TNBC cells by an NF-κB p65-dependent mechanism. We show that the MUC1-C→NF-κB p65→BCL2A1 pathway is of importance in the response to treatment with agents, such as ABT-737, that target other members of the BCL-2 family. In concert with these results, we show that targeting MUC1-C is an effective approach for downregulating BCL2A1 in ABT-737-resistant TNBC cells.

## Results

### MUC1-C induces BCL2A1 expression

**Fig. 1:**
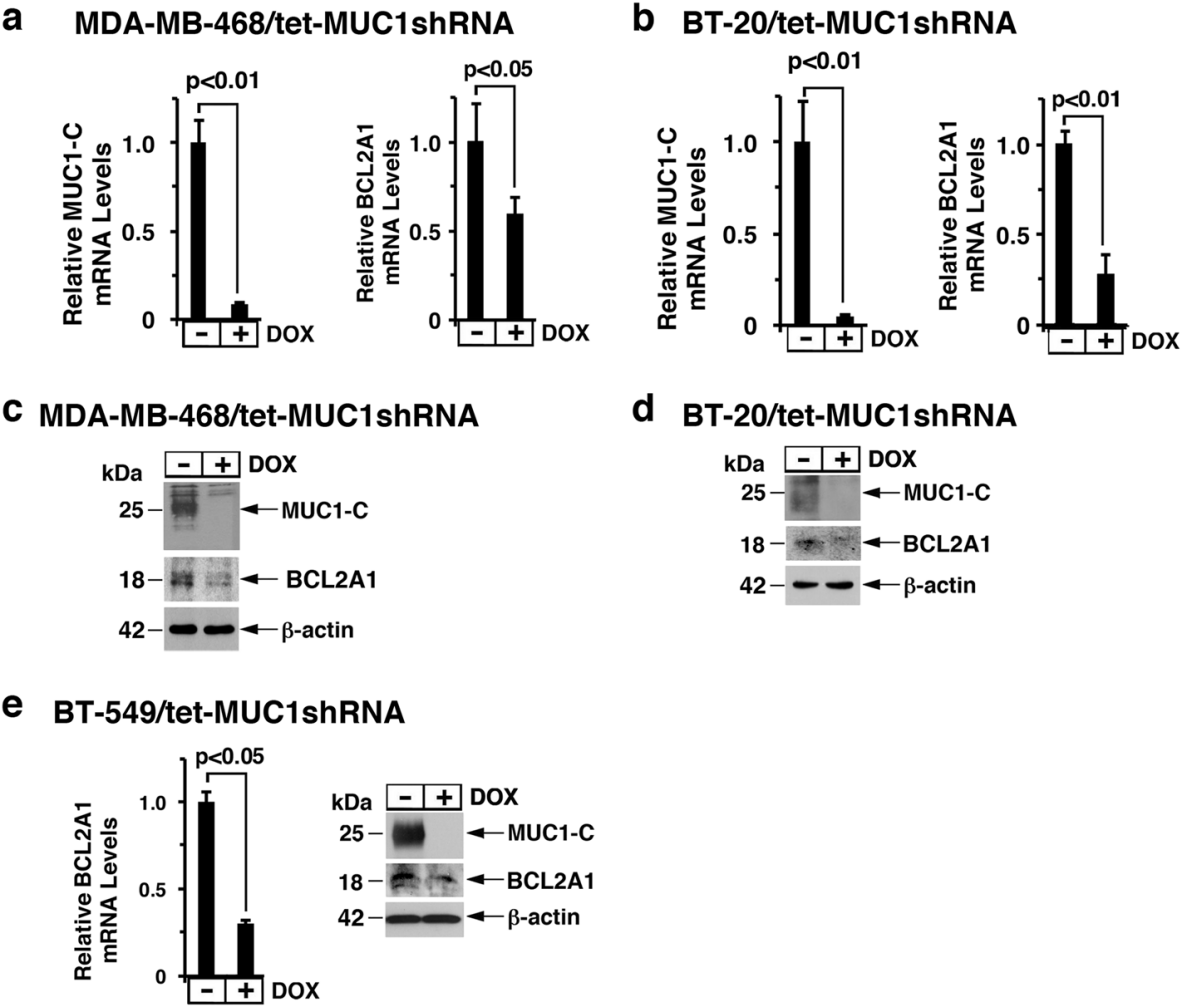
Downregulation of MUC1-C decreases BCL2A1 expression. **a**, **b** MDA-MB-468 (**a**) and BT-20 (**b**) cells were transduced to stably express tetracycline-inducible MUC1 shRNA (tet-MUC1shRNA). Cells treated with or without 500 ng/ml DOX for 3 d were analyzed for MUC1 (left) and BCL2A1 mRNA levels (right) by qRT–PCR. The results (mean ± SD of three determinations) are expressed as mRNA levels relative to those in control DOX-untreated cells (assigned a value of 1). **c**, **d** Lysates from cells treated with or without 500 ng/ml DOX for 10 d were immunoblotted with the indicated antibodies. **e** BT-549/tet-MUC1shRNA cells treated with or without 500 ng/ml DOX for 5 d were analyzed for BCL2A1 mRNA levels by qRT–PCR (left). The results (mean ± SD of three determinations) are expressed as mRNA levels relative to those in control DOX-untreated cells (assigned a value of 1). Lysates from cells treated with or without 500 ng/ml DOX for 10 d were immunoblotted with the indicated antibodies (right)

To assess the potential involvement of MUC1-C in the regulation of BCL2A1, we established MDA-MB-468 breast cancer cells stably expressing tetracycline-inducible control shRNA (tet-CshRNA) or MUC1 shRNA (tet-MUC1shRNA). Treatment of MDA-MB-468/tet-MUC1shRNA cells with doxycycline (DOX) was associated with suppression of MUC1-C and BCL2A1 mRNA levels (Fig. 1a, left and right). By contrast, DOX treatment of MDA-MB-468/tet-CshRNA cells had no significant effect on MUC1-C or BCL2A1 expression (Supplemental Fig. S1a, left and right). Similar results were obtained with BT-20 cells stably expressing tet-MUC1shRNA (Fig. 1b, left and right) or tet-CshRNA (Supplemental Fig. S1b, left and right) cells. In concert with these results, DOX treatment of MDA-MB-468/tet-MUC1shRNA (Fig. 1c) and BT-20/tet-MUC1shRNA (Fig. 1d) cells was associated with downregulation of BCL2A1 protein. BT-549/tet-MUC1shRNA cells also responded to DOX by suppressing BCL2A1 expression (Fig. 1e, left and right), confirming that MUC1-C drives BCL2A1 expression in TNBC cells.

### Targeting MUC1-C suppresses BCL2A1 expression

**Fig. 2:**
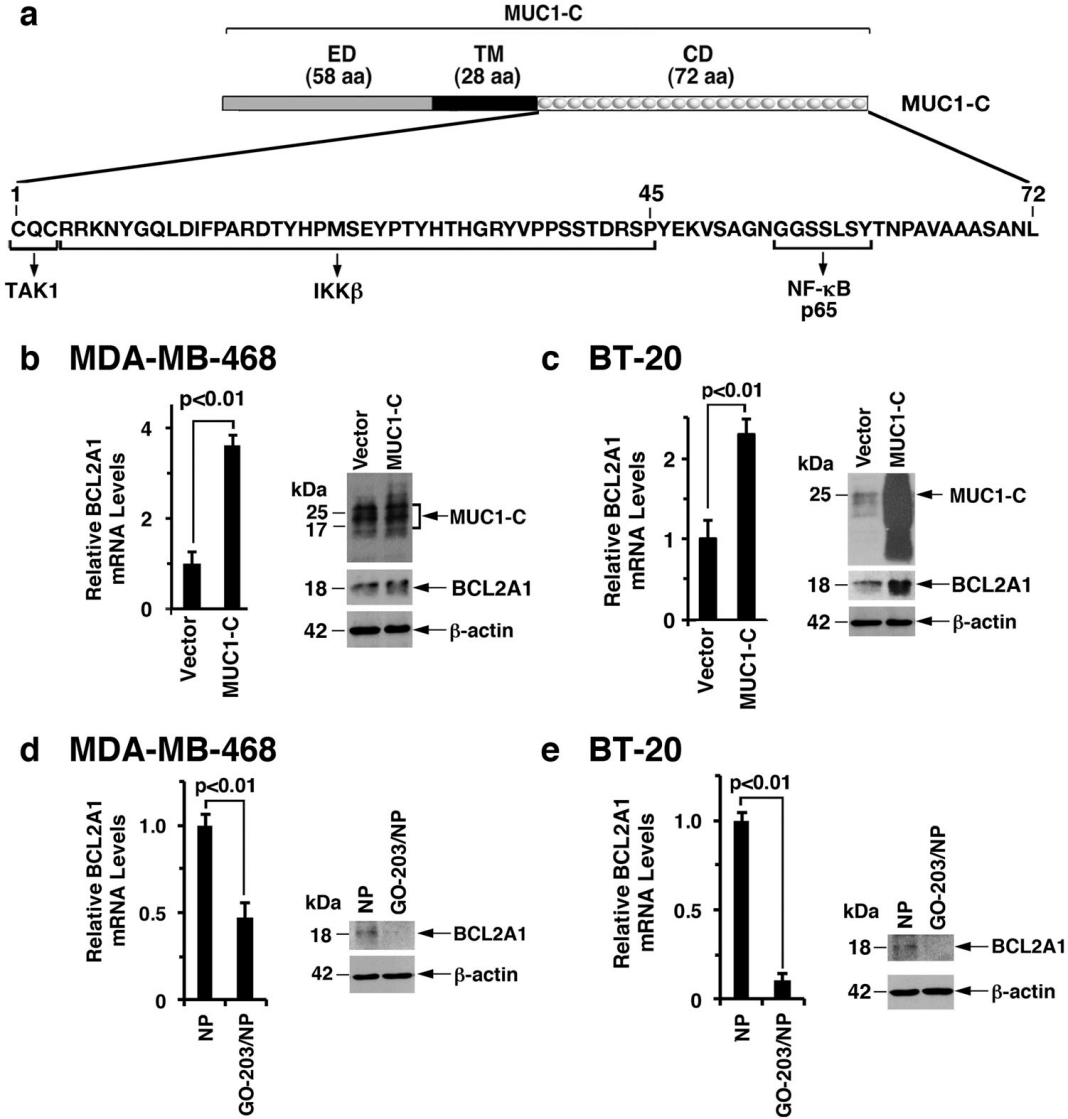
MUC1-C drives BCL2A1 expression. **a** Schema of the MUC1-C subunit with the sequence of the 72-amino acid intrinsically disordered cytoplasmic domain (CD). The CQC motif is required for MUC1-C homodimerization and is the target of GO-203. The MUC1-C cytoplasmic domain activates the inflammatory TAK1→IKK→NF-κB p65 pathway by direct interactions with these effectors. **b**, **c** MDA-MB-468 (**b**) and BT-20 (**c**) cells stably transduced to express a control or MUC1-C vector were analyzed for BCL2A1 mRNA levels by qRT–PCR. The results (mean ± SD of 3 determinations) are expressed as BCL2A1 mRNA levels relative to those in vector cells (assigned a value of 1) (left). Lysates were immunoblotted with the indicated antibodies (right). **d**, **e** MDA-MB-468 (**d**) and BT-20 (**e**) cells treated with empty NPs or 7.5 μM GO-203/NPs for 5 d were analyzed for BCL2A1 mRNA levels by qRT–PCR. The results (mean ± SD of three determinations) are expressed as BCL2A1 mRNA levels relative to those in cells treated with empty NPs (assigned a value of 1) (left). Lysates from cells treated with empty NPs or 7.5 μM GO-203/NPs for 7 d were immunoblotted with the indicated antibodies (right)

MUC1-C is a transmembrane protein with a 58-amino acid (aa) extracellular domain and a 72-aa intrinsically disordered cytoplasmic domain^23^ (Fig. 2a). The structure of the MUC1-C subunit is of importance in distinguishing MUC1-C from MUC1-N, which is devoid of a transmembrane domain and is shed from the cell surface. The MUC1-C CQC motif in the cytoplasmic domain is necessary for MUC1-C homodimerization and import to the nucleus^24,25^ (Fig. 2a). The MUC1-C cytoplasmic domain also interacts directly with IKKβ^5^ and NF-κB p65,^6^ thereby activating this inflammatory pathway. To extend the above observations, we stably overexpressed MUC1-C in MDA-MB-468 cells and observed the induction of BCL2A1 mRNA and protein levels (Fig. 2b, left and right). Similar results were obtained in BT-20 cells overexpressing MUC1-C (Fig. 2c, left and right), indicating that MUC1-C, and not the shed MUC1-N subunit, is necessary for this response. The GO-203 peptide, which targets the CQC motif and inhibits MUC1-C homodimerization (Fig. 2a),^23,25^ has been incorporated into polymeric nanoparticles (GO-203/NPs) for delivery into tumor cells.^26^ Treatment of MDA-MB-468 cells with GO-203/NPs was associated with the suppression of BCL2A1 mRNA and protein levels compared with empty NPs (Fig. 2d, left and right). We also found that targeting MUC1-C with GO-203/NPs effectively downregulated BCL2A1 expression in BT-20 cells (Fig. 2e, left and right). Of note, GO-203 blocks MUC1-C function^1^ and has no effect on MUC1-C expression in MDA-MB-468 and BT-20 cells.^19^

### MUC1-C promotes *BCL2A1* transcription by an NF-κB p65-mediated mechanism

**Fig. 3:**
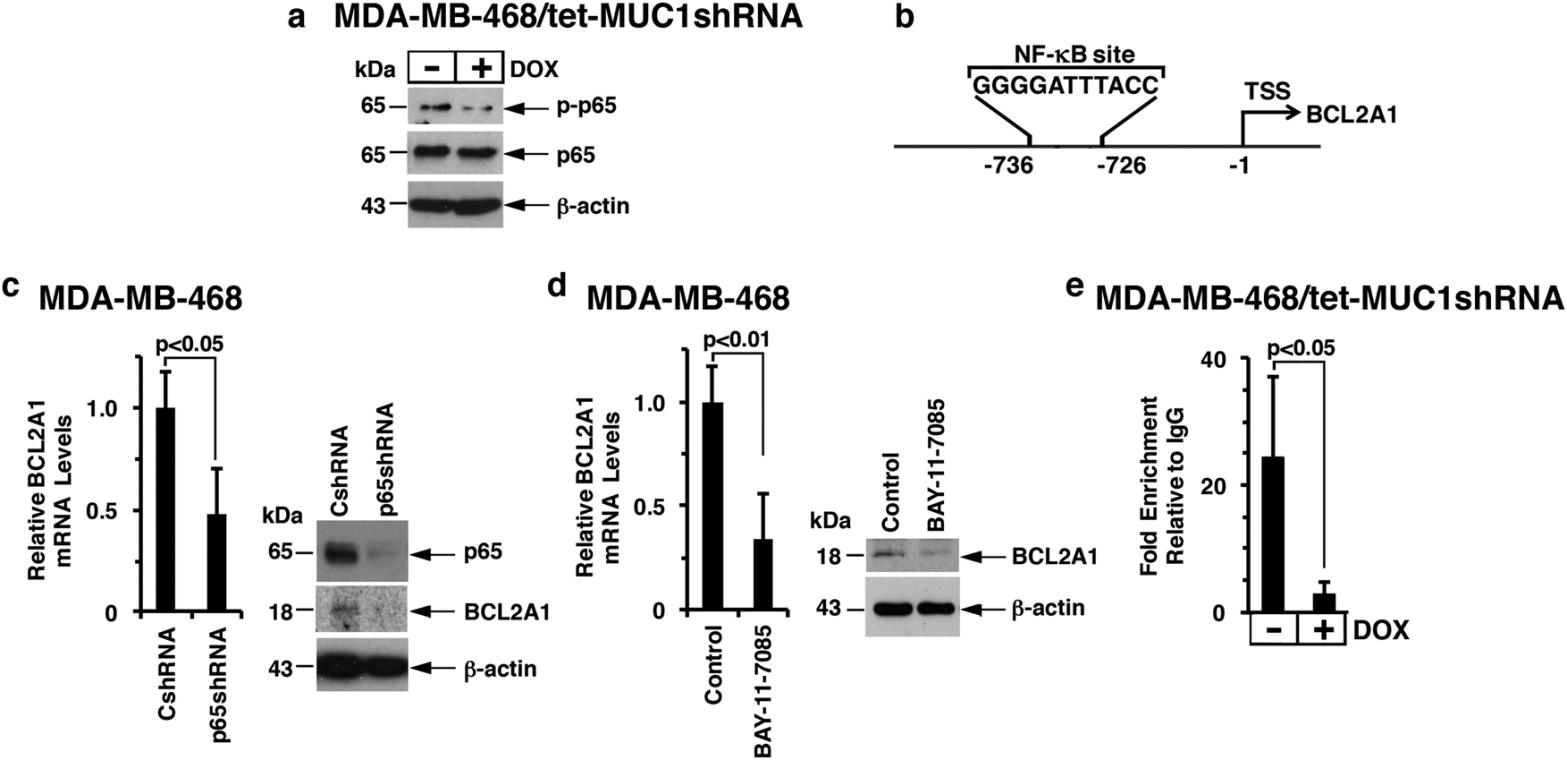
MUC1-C→NF-κB p65 signaling induces BCL2A1 expression. **a** Lysates from MDA-MB-468/tet-MUCshRNA cells treated with or without DOX for 7 d were immunoblotted with the indicated antibodies. **b** Schema of the BCL2A1 promoter with localization of the NF-κB binding motif at −736 to −726 upstream of the transcription start site (TSS). **c** MDA-MB-468 cells were stably transduced to express control shRNA (CshRNA) or NF-κB p65 shRNA (p65shRNA). Cells were analyzed for BCL2A1 mRNA levels by qRT–PCR. The results (mean ± SD of 3 determinations) are expressed as BCL2A1 mRNA levels relative to those in CshRNA cells (assigned a value of 1) (left). Lysates were immunoblotted with the indicated antibodies (right). **d** MDA-MB-468 cells treated with 5 μM BAY-11-7085 or vehicle control for 30 h were analyzed for BCL2A1 mRNA levels by qRT–PCR. The results (mean ± SD of three determinations) are expressed as BCL2A1 mRNA levels relative to those in control cells (assigned a value of 1) (left). Cell lysates were immunoblotted with the indicated antibodies (right). **e** Soluble chromatin from MDA-MB-468/tet-MUC1shRNA cells cultured with or without DOX for 5 d was precipitated with anti-NF-κB p65 or a control IgG. The final DNA samples were amplified by qPCR with primers targeting the NF-κB binding region in the BCL2A1 promoter. The results (mean ± SD of three determinations) are expressed as the relative fold enrichment compared with the IgG control (assigned a value of 1)

MUC1-C activates the inflammatory TAK1→IKK→NF-κB p65 pathway, binds directly to NF-κB p65 and promotes NF-κB p65-mediated induction of gene transcription.^4–6^ In this way, we found that DOX-induced silencing of MUC1-C in MDA-MB-468/tet-MUC1shRNA cells decreased phospho-NF-κB p65 (p-p65) (Fig. 3a). A similar response was obtained with DOX-treated BT-20/tet-MUC1shRNA cells (Supplemental Fig. S2a). The *BCL2A1* promoter contains a consensus motif for NF-κB p65 binding (Fig. 3b).^27^ Accordingly, we asked whether silencing NF-κB p65 in MDA-MB-468 cells is associated with the downregulation of *BCL2A1* transcription. Using this approach, we found that knockdown of NF-κB p65 decreased BCL2A1 mRNA and protein levels (Fig. 3c, left and right). In addition, treatment of MDA-MB-468 cells with BAY-11-7085, an inhibitor of IκBα phosphorylation, downregulated BCL2A1 expression (Fig. 3d, left and right). Similar results were obtained with BT-20 cells (Supplemental Fig. S2b). Further supporting the involvement of the MUC1-C→NF-κB p65→BCL2A1 pathway, MUC1-C silencing was associated with a decrease in NF-κB p65 occupancy at the *BCL2A1* promoter (Fig. 3e).

### Targeting MCL-1 induces BCL2A1 expression

**Fig. 4:**
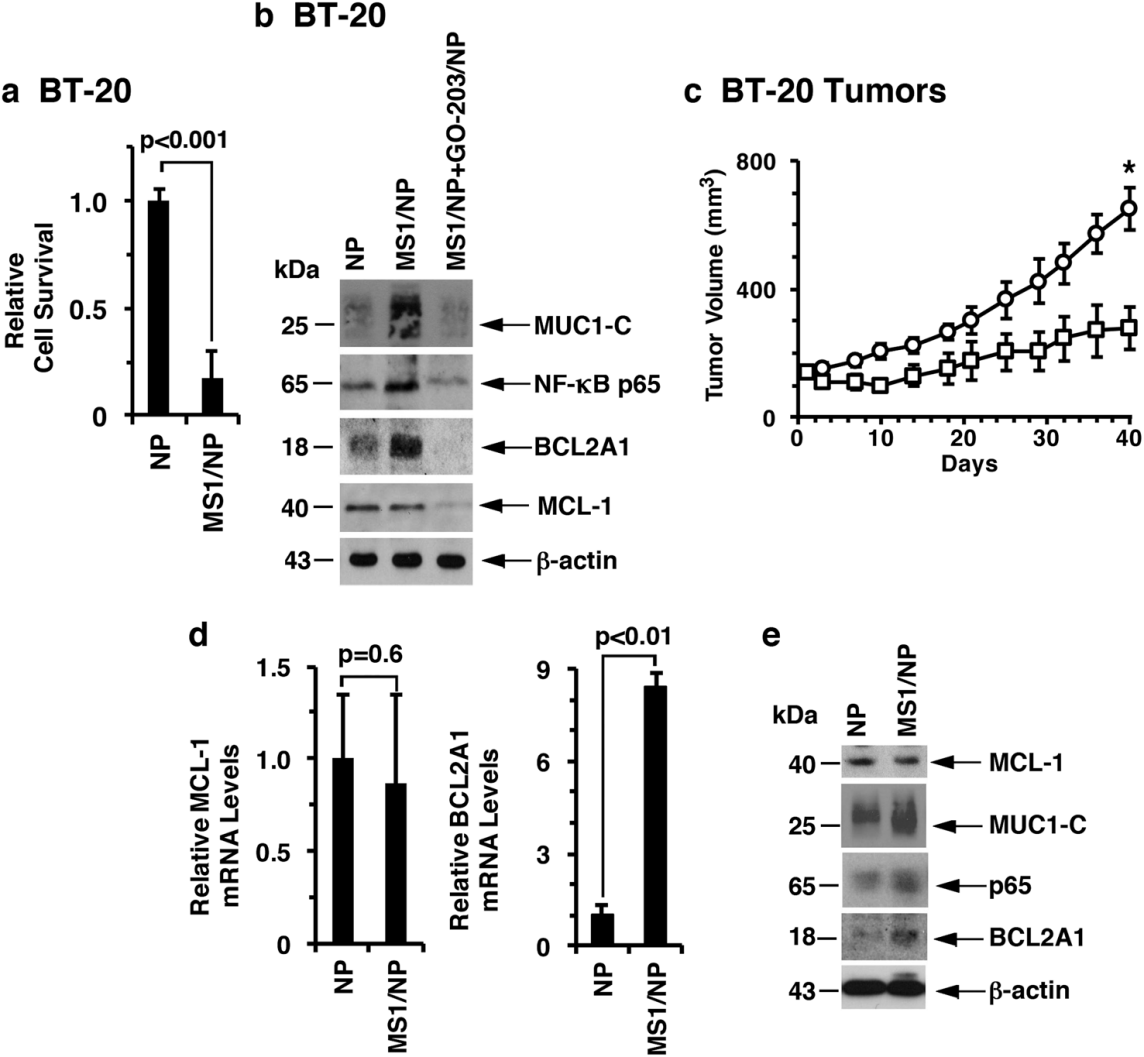
Targeting MCL-1 activates the MUC1-C→NF-κB p65→BCL2A1 pathway. **a** BT-20 cells were treated with empty NPs or 7.5 μM MS1/NPs for 5 d. The results are expressed as the relative survival compared with untreated cells (assigned a value of 1). **b** BT-20 cells were treated with (i) empty NPs, (ii) 7.5 μM MS1/NPs, or (iii) 3.75 μM MS1/NPs and 3.75 μM GO-203/NPs for 5 d. Lysates were immunoblotted with the indicated antibodies. **c** BT-20 cells were injected subcutaneously into the flanks of nude mice. Mice with established tumors were pair-matched and then treated intraperitoneally with empty NPs (circles) or 20 mg/kg MS1/NPs (squares) each week for 3 weeks. The results are expressed as the tumor volume (mean ± SEM; six mice per group). *p < 0.01. Blinding was not performed. Tumors were collected on day 40. **d** Tumor cells were analyzed for MCL-1 (left) and BCL2A1 (right) mRNA levels by qRT–PCR. The results (mean ± SD of three determinations) are expressed as the relative mRNA levels compared with empty NP-treated tumors (assigned a value of 1). **e** Lysates from empty NP- and MS1/NP-treated tumors were immunoblotted with the indicated antibodies

The above findings, taken together with those in our previous work,^19^ support a model in which MUC1-C regulates both MCL-1 and BCL2A1. To investigate the relationship between MUC1-C-induced MCL-1 and BCL2A1 expression, we targeted MCL-1 with the MS1 peptide, which binds to MCL-1 with a dissociation constant of ~2 nM and is >500-fold selective for MCL-1 over other BCL-2 family members.^28^ As described for GO-203/NPs,^26^ we encapsulated the MS1 peptide in NPs for intracellular delivery. Treatment of BT-20 and MDA-MB-468 cells with MS1/NPs, but not control NPs, was associated with cell the loss of survival (Fig. 4a and Supplemental Fig. S3). MCL-1 plays a role in suppressing the generation of reactive oxygen species (ROS).^29^ Treatment with MS1/NPs had little, if any, effect on MCL-1 expression and was associated with the upregulation of MUC1-C, NF-κB p65 and BCL2A1 (Fig. 4b), consistent with the finding that ROS activate the MUC1-C→NF-κB p65 pathway.^19^ Additionally, combining MS1/NPs with GO-203/NPs to inhibit MUC1-C signaling abrogated the MS1/NP-induced increases in NF-κB p65 and BCL2A1 (Fig. 4b), supporting the importance of MUC1-C in linking MCL-1 activity and the MUC1-C→NF-κB p65→BCL2A1 axis (Fig. 4b). In concert with our previous findings,^19^ targeting MUC1-C with GO-203/NPs was also associated with the downregulation of MCL-1 expression (Fig. 4b). GO-203/NPs are effective against TNBC xenografts;^26^ however, MS1/NPs have not been evaluated in vivo. Accordingly, we administered MS1/NPs to mice bearing established BT-20 tumors. The results showed that MS1/NP treatment was effective at significantly inhibiting BT-20 tumor growth (Fig. 4c). In addition, targeting MCL-1 in BT-20 tumors was associated with marked increases in BCL2A1 mRNA levels (Fig. 4d) and the upregulation of MUC1-C, NF-κB p65 and BCL2A1 (Fig. 4e), indicating that targeting MCL-1 function in the absence of changes in expression induces cell death and compensatory activation of the MUC1-C→NF-κB p65→BCL2A1 pathway.

### Resistance to ABT-737 and ABT-263 is associated with activation of the MUC1-C→NF-κB→BCL2A1 pathway

**Fig. 5:**
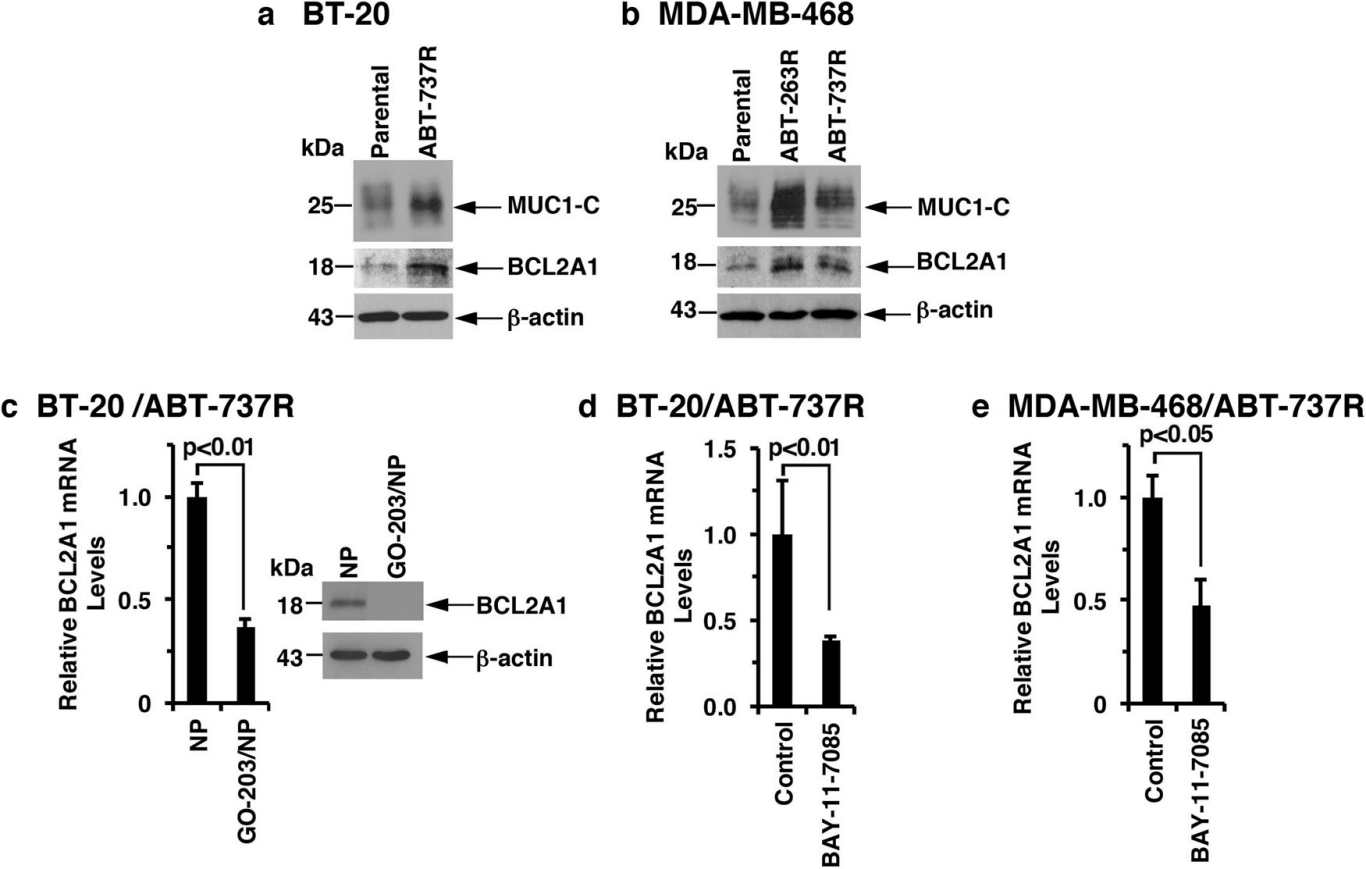
MUC1-C→NF-κB signaling upregulates BCL2A1 in ABT-737-resistant cells. **a** Lysates from BT-20 (parental) and BT-20/ABT-737R cells were immunoblotted with the indicated antibodies. **b** Lysates from MDA-MB-468 (parental) and the respective ABT-resistant cells were immunoblotted with the indicated antibodies. **c** BT-20/ABT-737R cells were treated with empty NPs or 2.5 μM GO-203/NPs for 5 d. Cells were analyzed for BCL2A1 mRNA levels by qRT–PCR. The results (mean ± SD of three determinations) are expressed as BCL2A1 mRNA levels relative to those in empty NP-treated cells (assigned a value of 1) (left). Lysates were immunoblotted with the indicated antibodies (right). **d**, **e** BT-20/ABT-737R (**d**) and MDA-MB-468/ABT-737R (**e**) cells treated with 5 μM BAY-11-7085 or vehicle control for 12 h were analyzed for BCL2A1 mRNA levels by qRT–PCR. The results (mean ± SD of three determinations) are expressed as BCL2A1 mRNA levels relative to those in control cells (assigned a value of 1)

The small molecules ABT-737 and ABT-263 target BCL-2, BCL-xL and BCL-W but not MCL-1 or BCL2A1.^30,31^ Selection of BT-20 and MDA-MB-468 cells for resistance to ABT-737 (ABT-737R) is associated with the upregulation of MUC1-C and MCL-1.^19^ We also found that BCL2A1 levels were upregulated in BT-20/ABT-737R cells (Fig. 5a). In concert with these results, MDA-MB-468 cells resistant to ABT-737 (MDA-MB-468/ABT-737R) or ABT-263 (MDA-MB-468/ABT-263R) also exhibited upregulation of BCL2A1 (Fig. 5b). To determine whether MUC1-C also drives BCL2A1 expression in the setting of ABT-737 resistance, we treated BT-20/ABT-737R cells with GO-203/NPs. As was reported for MCL-1,^19^ targeting MUC1-C was clearly associated with the downregulation of BCL2A1 expression (Fig. 5c). However, in contrast to MCL-1, which is stabilized by MUC1-C-induced signaling,^19^ targeting MUC1-C in BT-20/ABT-737R cells decreased BCL2A1 mRNA and protein levels (Fig. 5c), supporting a potential transcriptional mechanism. Indeed, inhibiting NF-κB p65 with BAY-11-7085 in BT-20/ABT-737R (Fig. 5d) and MDA-MB-468/ABT-737R (Fig. 5e) cells effectively suppressed BCL2A1 mRNA levels.

### Targeting MUC1-C is effective in the setting of ABT-737 resistance

**Fig. 6:**
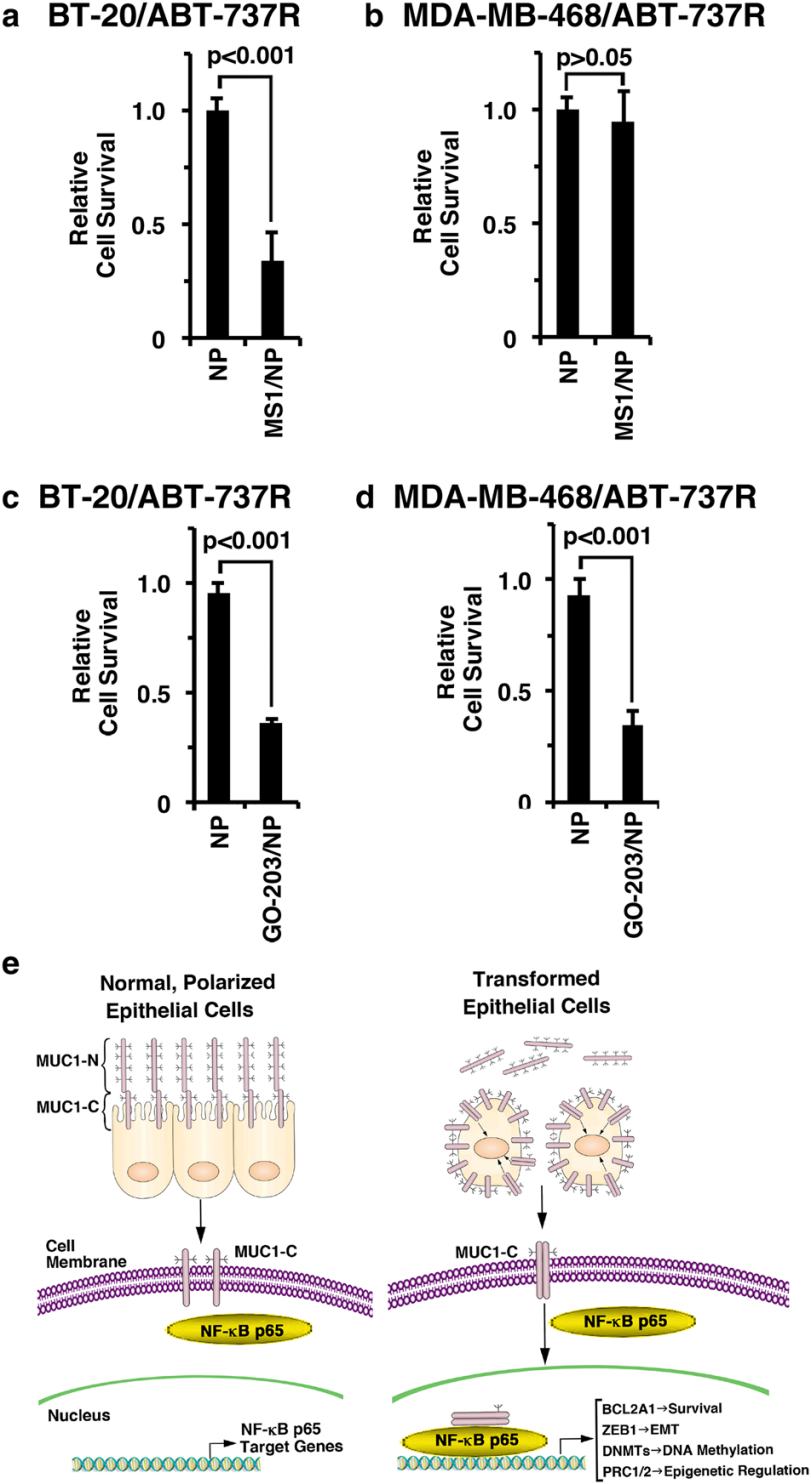
Targeting MUC1-C is effective against ABT-resistant cells with BCL2A1 overexpression. **a**, **b** BT-20/ABT-737R (**a**) and MDA-MB-468/ABT-737R (**b**) cells were treated with empty NPs or 7.5 μM MS1/NPs for 7 d. The results are expressed as relative survival compared to untreated cells (assigned a value of 1). **c**, **d** BT-20/ABT-737R (**c**) and MDA-MB-468/ABT-737R (**d**) cells were treated with empty NPs or 7.5 μM GO-203/NPs for 7 d. The results are expressed as relative survival compared to untreated cells (assigned a value of 1). **e** Schema depicting the function of the MUC1-C→NF-κB p65 pathway in integrating the induction of BCL2A1 expression with the EMT program^7^ and epigenetic regulation^11,34,36^

Treatment of BT-20/ABT-737R cells with MS1/NPs was associated with the loss of survival (Fig. 6a). Interestingly, however, MDA-MB-468/ABT-737R cells were unaffected by MS1/NP exposure (Fig. 6b), consistent with the finding that BT-20 cells, but not MDA-MB-468 cells, are dependent on MCL-1 for survival.^32^ Notably, in contrast, BT-20/ABT-737R (Fig. 6c) and MDA-MB-468/ABT-737R (Fig. 6d) cells were both sensitive to treatment with GO-203/NPs, supporting our findings that targeting MUC1-C effectively downregulates MCL-1 and BCL2A1.

## Discussion

TNBC is often a clinically aggressive disease.^33^ In addition, options for TNBC treatment have been limited by a lack of actionable molecular targets.^33^ The MUC1-C oncoprotein is aberrantly expressed in most TNBCs^1,2^ and has been linked to diverse aspects of TNBC progression, including the downregulation of cell polarity factors and the induction of EMT (Fig. 6e).^7–9^ The present study demonstrates that targeting MUC1-C in TNBC cells with genetic and pharmacological approaches suppresses the expression of the anti-apoptotic BCL2A1 protein. *BCL2A1* has been identified as an oncogene in melanoma and B-cell malignancies;^21^ however, there are no reports on a role for BCL2A1 in breast cancer cells. In this regard, the upregulation of *BCL2A1* in breast cancers is associated with a significant decrease in relapse-free survival (Supplemental Fig. S4). Our results show that MUC1-C activates *BCL2A1* expression by an NF-κB p65-mediated mechanism. In this way, MUC1-C binds directly to NF-κB p65 and activates NF-κB target genes, including *BCL-xL* and *MUC1* itself.^6^ By extension, the present results show that MUC1-C similarly increases NF-κB p65 occupancy at the *BCL2A1* promoter and upregulates BCL2A1 expression (Fig. 6e). Of note, these findings do not exclude a potential role for MUC1-C in the post-transcriptional control of BCL2A1 expression, which is at present an undeveloped area of investigation. MUC1-C also induces ZEB1 by increasing NF-κB p65 occupancy at the *ZEB1* promoter.^6,7^ In turn, ZEB1 suppresses certain genes, such as *miR-200c*, that promote polarity and thereby drives EMT.^7^ In addition, the inflammatory MUC1-C→NF-κB p65 pathway has been linked to the epigenetic regulation of gene expression in TNBC cells by (i) driving *DNMT1* and *DNMT3b* and, in turn, alterations in DNA methylation patterns,^11^ (ii) upregulating components of the Polycomb Repressive Complex 1 (PRC1) and derepressing *HOX* genes,^34,35^ and (iii) inducing *EZH2* in association with PRC2 activation and the repression of tumor suppressor genes^36,37^ (Fig. 6e). Previous and present findings support a model in which MUC1-C→NF-κB p65 signaling integrates the induction of the anti-apoptotic BCL2A1 pathway with EMT and epigenetic reprogramming in TNBC cells (Fig. 6e).

Aberrant expression of MCL-1 is important for TNBC cell survival.^32^ As found here for BCL2A1, targeting MUC1-C in TNBC cells is associated with the downregulation of MCL-1.^19^ However, in contrast to MUC1-C-induced *BCL2A1* expression by NF-κB p65, MUC1-C increases MCL-1 levels by activating the PI3K→AKT and MEK→ERK pathways, thereby stabilizing MCL-1 protein.^19^ To the best of our knowledge, there is no known relationship between MCL-1 and BCL2A1; nonetheless, the finding that MUC1-C regulates the expression of both proteins invokes the potential for cross-talk. To search for such evidence, we targeted MCL-1 with the highly potent and specific MS1 peptide,^28^ which was encapsulated in nanoparticles for cell delivery. Interestingly, we found that targeting MCL-1 was associated with the upregulation of MUC1-C, NF-κB p65 and BCL2A1. Moreover, the induction of BCL2A1 expression was mediated by a MUC1-C-dependent mechanism. MCL-1 protects TNBC cells from death in response to the BH3 mimetics ABT-737 and ABT-263.^30,31^ Additionally, the selection of TNBC cells for resistance to ABT-737 and ABT-263 is associated with the upregulation of MUC1-C and thereby MCL-1.^19^ Consistent with the observations that MUC1-C drives both MCL-1 and BCL2A1 expression, the present results further demonstrate that MUC1-C induces BCL2A1 expression in the setting of resistance to ABT-737/263. These findings may have clinical relevance in that certain TNBCs overexpressing MCL-1 and BCL2A1 could exhibit resistance to MCL-1 inhibitors that are under development.^38–40^ In this context, we found that BT-20/ABT-737R cells, which overexpress MCL-1 and BCL2A1, were sensitive to targeting MCL-1 with MS1/NPs. By contrast, MDA-MB-468/ABT-737R cells, which also overexpress MCL-1 and BCL2A1, were resistant to targeting MCL-1. This discrepancy in sensitivity to targeting MCL-1 is explained, at least in part, by the demonstration that BT-20 cells, but not MDA-MB-468 cells, are dependent on MCL-1 for survival.^32^

Our findings further demonstrate that targeting MUC1-C with GO-203/NPs effectively downregulates both MCL-1^19^ and BCL2A1 in drug-naïve and ABT-737-resistant TNBC cells. In contrast to the differential effects of MS1/NPs, we found that both BT-20/ABT-737R and MDA-MB-468/ABT-737R cells were sensitive to targeting MUC1-C, indicating that treatment with GO-203/NPs may be an attractive option in the setting of ABT-737 or ABT-263 resistance. Obatoclax, a pan-BCL-2 family inhibitor that targets MCL-1 and BCL2A1,^21,41^ is being evaluated in early-phase clinical trials in combination with cytotoxic and targeted agents (ClinicalTrials.Gov). However, to our knowledge, there are no selective BCL2A1 inhibitors in the clinic. In this context and regarding the translational relevance of the present findings, GO-203 has completed Phase I evaluation in patients with advanced solid tumors, demonstrating an acceptable safety profile and clinical activity. GO-203 has a circulating half-life of 5–7 h, which requires daily drug delivery to maintain necessary tumor exposure levels. To circumvent this challenge, GO-203 has been formulated in NPs for sustained delivery, such that weekly administration of GO-203/NPs to mice bearing syngeneic or xenograft tumors is as active as daily dosing with GO-203.^26^ Based on the present findings, the GO-203/NP formulation is under development for the treatment of patients with refractory, drug-resistant TNBC.

## Materials and methods

### Cell culture

Human MDA-MB-468 cells were grown in Dulbecco’s modified Eagle’s medium (DMEM) (Corning, Manassas, VA, USA). BT-20 cells were grown in Eagle's Minimum Essential Medium (EMEM) (ATCC, Manassas, VA, USA). BT-549 cells were cultured in RPMI1640 medium (ATCC). Media were supplemented with 10% heat-inactivated fetal bovine serum (HI-FBS), 100 U/ml penicillin and 100 μg/ml streptomycin. Cells were infected with lentiviral vectors to stably express scrambled control shRNA (CshRNA; Sigma, St. Louis, MO, USA), NF-κB p65 shRNA (Sigma), or MUC1-C.^19^ Cells were treated with (i) the NF-κB inhibitor BAY-11-7085 (Millipore, Billerica, MA, USA) or DMSO as the vehicle control, (ii) the MUC1-C inhibitor GO-203 or the control CP-2 peptide,^34^ or (iii) empty nanoparticles (NPs), GO-203/NPs^26^ or MS1/NPs.^28^ Authentication of the cells was performed by short tandem repeat (STR) analysis. Cells were monitored for mycoplasma contamination using the MycoAlert® Mycoplasma Detection Kit (Lonza, Rockland, MA, USA).

### Tetracycline-inducible MUC1 silencing

MUC1shRNA (MISSION shRNA; Sigma, TRCN0000122938), which targets the MUC1-C sequence ACAGACTTCAATAGTATAA, and a control scrambled CshRNA (Sigma) were inserted into the pLKO-tet-puro vector (Addgene, Cambridge, MA, USA; Plasmid #21915). The viral vectors were produced in HEK293T cells as previously described.^19,34^ Cells were selected for growth in 1–3 μg/ml puromycin. Cells were treated with doxycycline (DOX; Sigma).

### RNA extraction and real-time quantitative reverse transcription PCR (qRT–PCR)

Total RNA was isolated with Trizol reagent (Invitrogen, Carlsbad, CA, USA) following the manufacturer’s protocol. The High Capacity cDNA Reverse Transcription kit (Applied Biosystems, Grand Island, NY, USA) was used to synthesize complementary DNA from 2.0 μg of total RNA as described previously.^34^ Power SYBR Green PCR Master Mix (Applied Biosystems) was used with 1 μl of diluted cDNA for each sample. The samples were amplified using the 7300 Real-time PCR System (Applied Biosystems). Primers used for qRT–PCR are listed in Supplemental Table S1.

### Chromatin immunoprecipitation (ChIP) assay

Soluble chromatin was isolated from 5 × 10^6^ cells and immunoprecipitated with anti-NF-κB p65 and a control non-immune IgG (Santa Cruz Biotechnology, Dallas, TX, USA). For real-time ChIP qPCR, the SYBR green system was used with the ABI Prism 7300 sequence detector (Applied Biosystems). The data are reported as the relative fold enrichment.^11^ The primers used for ChIP qPCR of the *BCL2A1* promoter are listed in Supplementary Table S2.

### Immunoblot analysis

Western blot analysis was performed as described previously.^34^ Whole cells were lysed in NP-40 buffer containing a phosphatase inhibitor and protease inhibitor cocktail. Immunoblotting was performed with anti-MUC1-C (NeoMarkers, Fremont, CA, USA), anti-phospho-p65(Ser-536), anti-NF-κB p65 (Santa Cruz Biotechnology), anti-BCL2A1 (Cell Signaling Technology, Danvers, MA, USA) and anti-β-actin antibodies (Sigma).

### Cell survival assays

Cell survival was determined using the Alamar Blue Cell Viability Assay according to the manufacturer’s protocol (ThermoFisher Scientific, Waltham, MA, USA).

### TNBC tumor xenograft studies

BT-20 cells (5 × 10^6^) were injected subcutaneously into the flanks of 6-week-old female nu/nu mice. After tumors reached ~100 mm^3^, mice were pair-matched in two groups and treated intraperitoneally with empty NPs or 20 mg/kg MS1/NPs each week for 3 weeks. Tumor volume was calculated as *V* = *L* × *W*^2^/2, where *L* and *W* are the larger and smaller diameters, respectively.

### Statistical analysis

Each experiment was repeated at least three times. Data are expressed as the mean ± SD or SEM. The unpaired Student’s t-test was used to examine the differences between the means of two groups. A *p*-value < 0.05 indicated a statistically significant difference.

## Electronic supplementary material

Supplemental Material
