# Supplementary material for: Targeting MUC1-C suppresses BCL2A1 in triple-negative breast cancer
Source: Signal Transduct Target Ther. 2018 May 12;3:13. doi: 10.1038/s41392-018-0013-x (PMC5948210; doi:10.1038/s41392-018-0013-x)
Supplement: Supplementary file 1 — Supplemental Material [file 41392_2018_13_MOESM1_ESM.pdf]

Supplemental Material

Supplemental Tables S1-S2 and Figures S1-S4

TARGETING MUC1-C SUPPRESSES BCL2A1 IN TRIPLE-NEGATIVE BREAST CANCER

Masayuki Hiraki[1*], Takahiro Maeda[1], Caining Jin, Maroof Alam, Audrey Bouillez, Tsuyoshi Hata, Ashujit Tagde, Amy Keating# and Donald Kufe

Dana-Farber Cancer Institute
Harvard Medical School
Boston, MA
and
#Departments of Biology and Biological Engineering
Massachusetts Institute of Technology
Cambridge, MA

[1]**Equal contribution.**

Supplemental Table S1. Primers used for qRT–PCR

GAPDH forward primer: 5′-CCATGGAGAAGGCTGGGG-3′
GAPDH reverse primer: 5′-CAAAGTTGTCATGGATGACC-3′
MUC1-C forward primer: 5′-TACCGATCGTAGCCCCTATG-3′
MUC1-C reverse primer: 5′-CTCACCAGCCCAAACAGG-3′
BCL2A1 forward primer: 5′-TTACAGGCTGGCTCAGGACT-3′
BCL2A1 reverse primer: 5′-AGCACTCTGGACGTTTTGCT-3′
MCL-1 forward primer: 5′-TGCTGGAGTTGGTCGGGGAA-3′
MCL-1 reverse primer: 5′-TCGTAAGGTCTCCAGCGCCT-3′

Supplemental Table S2. Primers used for ChIP qPCR

BCL2A1 forward primer: 5′-ACTGCAACTTCTGTCTCCCG-3′
BCL2A1 reverse primer: 5′-AGCACTTGGGAGGACAAGG-3′

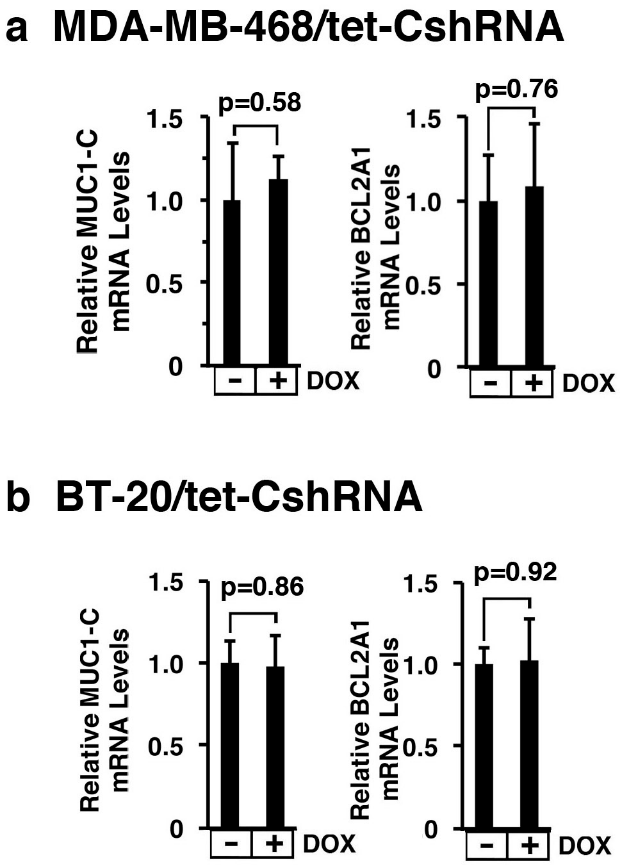

Supplemental Figure S1. Effects of DOX on control MDA-MB-468/tet-CshRNA and BT-20/tet-CshRNA cells. a and b. MDA-MB-468 (a) and BT-20 (b) cells were transduced to stably express a tetracycline-inducible Control shRNA (tet-CshRNA). Cells treated with or without 500 ng/ml DOX for 3 d were analyzed for MUC1-C (left) and BCL2A1 mRNA levels (right) by qRT-PCR. The results (mean±SD of 3 determinations) are expressed as relative mRNA levels compared with that obtained for control DOX-untreated cells (assigned a value of 1).

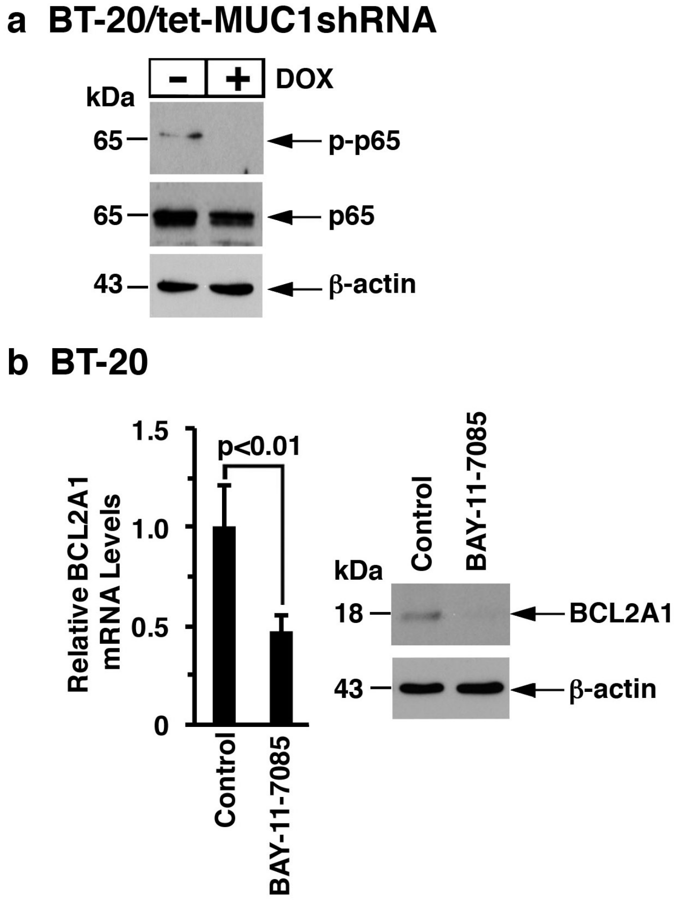

Supplemental Figure S2. MUC1-C activates NF-κB p65 and BCL2A1 expression in BT-20 cells. a. Lysates from BT-20/tet-MUCshRNA cells treated with or without DOX for 7 d were immunoblotted with the indicated antibodies. b. BT-20 cells treated with 5 μM BAY-11-7085 or vehicle control for 24 h were analyzed for BCL2A1 mRNA levels by qRT-PCR (mean±SD of 3 determinations) (left). Cell lysates were immunoblotted with the indicated antibodies (right).

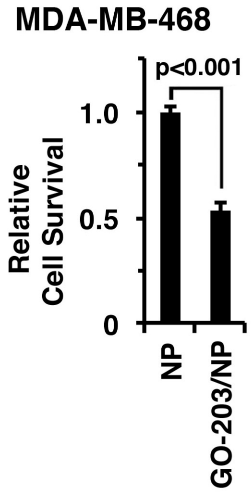

Supplemental Figure S3. Targeting MCL-1 inhibits survival in MDA-MB-468 cells. MDA-MB-468 cells were treated with empty NPs or 7.5 μM MS1/NPs for 7 d. The results are expressed as relative survival compared to that obtained with untreated cells (assigned a value of 1).

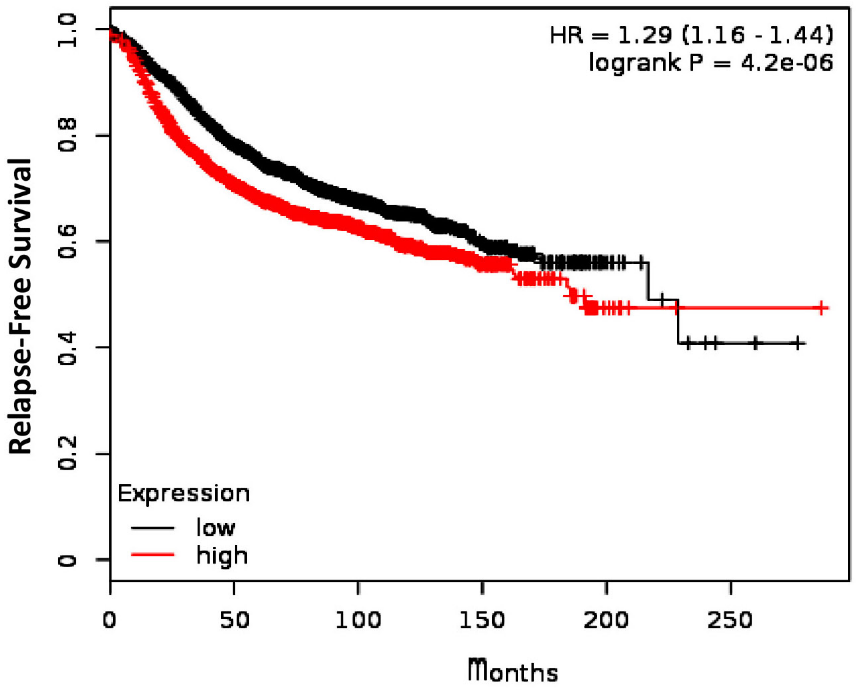

Supplemental Figure S4. Kaplan-Meier analysis of the probability of survival as a function of relative expression of BCL2A1 (205681_at). A cohort of 3951 breast cancer patients was analyzed from the KM plotter website. Patients were stratified with high (red) or low (blue) expression against the median. The plots were generated using www.kmplot.com (PMID: 20020197). The relapse-free survival curves were compared using the log-rank test. HR, hazard ratio. $P<0.05$ represents statistical significance.
